# Diacerein Downregulates *Nfkb*, Induces ALP Activity and Inhibits Osteoblast Apoptosis in Alveolar Bone of Rats with Periodontitis

**DOI:** 10.3390/biomedicines14020306

**Published:** 2026-01-29

**Authors:** Paulo Sérgio Cerri, Lucas de Andrade Rodrigues, Lays Cristina Gouvea, Gabriella de Oliveira, Estela Sasso-Cerri

**Affiliations:** Araraquara—Laboratory of Histology and Embryology—Department of Morphology, Genetics, Orthodontics and Pediatric Dentistry, School of Dentistry, São Paulo State University (UNESP), Araraquara 14801-903, SP, Brazil; la.rodrigues@unesp.br (L.d.A.R.); lays.gouvea@unesp.br (L.C.G.); gabriella.oliveira00@unesp.br (G.d.O.); estela.sasso@unesp.br (E.S.-C.)

**Keywords:** alveolar bone, periodontitis, osteoblasts, diacerein, apoptosis

## Abstract

**Objectives**: We evaluated the effect of diacerein, an anti-inflammatory drug, on the activity and survival of alveolar bone osteoblasts in rats with periodontitis. **Methods**: The rats with periodontitis received diacerein (PDG) or saline solution (PSG) for 7, 15 and 30 days. In gingiva samples, *Nfkb1* and *Bmp2* gene expressions were evaluated, and maxillae were processed for light and transmission electron microscopy. **Results**: In PDG, the tumor necrosis factor alpha (TNF-α) and interleukin-1 beta (IL-1β) immunoexpression decreased in parallel with the increase in alkaline phosphatase (ALP) and bone area over time. At 15 and 30 days, *Nfkb1* expression decreased in PDG compared to PSG, whereas at 30 days, the *Bmp2* expression was greater in PDG than in PSG. Immunofluorescence for IL-10, an anti-inflammatory cytokine, was greater in PDG than in PSG at 15 and 30 days. In PSG, the significant increase in the number of TUNEL-positive osteoblasts was accompanied by the presence of osteoblasts with condensed chromatin nuclei or caspase-3-immunolabelled osteoblasts. In contrast, the number of TUNEL-positive osteoblasts was significantly lower in PDG than in PSG specimens at all time points. **Conclusions**: Therefore, the diacerein-induced TNF-α and IL-1β inhibitory effect caused *Nfkb1* downregulation and, hence, prevented apoptosis in osteoblasts. The increased ALP activity and IL-10 in PDG indicate that diacerein mitigates periodontitis impact on alveolar bone in rat molars.

## 1. Introduction

Periodontitis is a chronic and multifactorial inflammatory disease characterized by alveolar bone resorption, resulting in interaction between bacterial products of the dysbiotic dental biofilm and the immune system, in addition to environmental and systemic factors [1,2,3]. The bacteria of the red complex (*Porphyromonas gingivalis*, *Tannerella forsythia*, and *Treponema denticola*) are primarily responsible for stimulating the production of pro-inflammatory cytokines, such as interleukin-1 beta (IL-1β) and tumor necrosis factor alpha (TNF-α), by macrophages and lymphocytes [3,4]. These cytokines stimulate the expression of other mediators such as matrix metalloproteinases (MMPs), receptor activator of nuclear factor kappa B (RANK) and its ligand (RANKL), and transcription factors such as nuclear factor kappa B (NF-κB), leading to tissue destruction and death of cells, especially through apoptosis [5]. It is known that the combined activation of these molecules increases osteoclast activity and inhibits osteogenic factors and osteoblast differentiation, favoring pathological alveolar bone resorption and potential tooth loss [4,6].

Osteoblasts are cuboidal-shaped bone cells located on the bone surface and are responsible for depositing bone matrix, called osteoid [7,8]. Moreover, osteoblasts participate in the mineralization process because they release several molecules, such as alkaline phosphatase (ALP). ALP is an enzyme that hydrolyses inorganic phosphate from the pyrophosphate (PPi) molecule, releasing phosphate (Pi) and allowing it to bind to calcium, contributing to the formation of hydroxyapatite crystals [9]. Osteoblast differentiation depends on transcription factors such as Runt-related transcription factor 2 (Runx2), Osterix (OSX), and β-catenin [10].

OSX, also known as Sp7, is a transcription factor expressed in the osteoblast nucleus and, like RUNX2, is responsible for the expression of osteogenic genes, among them are type I collagen, osteopontin, osteocalcin, bone sialoprotein, and osteonectin [11]. OSX^−^/^−^ mice show the presence of osteoblast progenitors due to RUNX2’s compensatory action, but these cells are unable to differentiate into mature osteoblasts, thereby preventing bone formation [12]. In addition to transcription factors, osteoblast formation is regulated by anti-osteoblastogenic cytokines, such as IL-1β and TNF-α, and interleukin-10 (IL-10) and bone morphogenetic protein 2 (BMP2), which stimulate the osteoblast activity [4]. While IL-1β and TNF-α inhibit differentiation and promote osteoblast apoptosis, hindering bone formation, IL-10 promotes bone formation through activation of the p38 MAPK pathway, and BMP2 through the SMAD pathway [4,13].

Diacerein is an anti-inflammatory drug that inhibits IL-1β and TNF-α and is used for the treatment of osteoarthritis, due to its beneficial effects on pain reduction and joint preservation [14,15,16]. Diacerein inhibits the synthesis of caspase-1 (ICE), which converts IL-1 into its active form, thus impairing the activation of this interleukin, in addition to reducing its interaction with the IL-1 receptor [17,18]. Beyond this action, several studies have shown that diacerein and its active metabolite rhein suppress the synthesis and release of TNF-α [19,20], as well as IL-1β-induced nitric oxide production, thereby attenuating the cytokine-mediated inflammatory response [16,17,21,22]. This effect is partly mediated by the inhibition of NF-κB activation [23], a transcription factor that drives the expression of pro-inflammatory mediators, including TNF-α, IL-1β and IL-6 [24,25]. Under physiological conditions, NF-κB remains bound to its inhibitor IκB, preventing its translocation to the nucleus. Activation of IKK promotes IκB phosphorylation and degradation, increasing NF-κB availability and inducing the transcription of inflammatory genes [26]. By preventing IκB degradation through IL-1 inhibition, diacerein limits NF-κB activation and consequently downregulates the expression of TNF-α, IL-1β, and IL-6 [16,17,19,21,22].

An in vivo study has demonstrated that diacerein has beneficial effects on the inflammatory condition of periodontitis by reducing the expression of IL-1β and TNF-α, which leads to decreased osteoclast differentiation and induces osteoclast apoptosis [6]. The inhibitory effect of diacerein on IL-1β and TNF-α also promotes the reduction of secondary mediators such as MMP-8 [27]. However, the exact effect of this drug on osteoblast formation and activity remains uncertain. Diacerein and rhein added to the culture of osteoarthritis (OA) subchondral osteoblasts reduced the osteocalcin release [28]. In contrast, it has been demonstrated that this drug increases β-catenin expression by OA osteoblasts [29], an important factor in the Wnt signaling pathway, which stimulates osteoblast differentiation and function [30]. Nevertheless, the main cytokine targets of diacerein, IL-1β and TNF-α, are known to have an inhibitory effect on osteoblasts. IL-1β treatment impairs osteoblast differentiation in vitro by activating the NF-κB and MAPK pathways, along with decreased expression of ALP, RUNX2 and OSX [31]. TNF-α downregulates RUNX2 and OSX expression at the transcriptional level by reducing their mRNA levels [32,33]. Additionally, TNF-α and IL-1β significantly increase FAS-mediated apoptosis in primary culture of human osteoblasts [13]. Because diacerein has shown a potent inhibitory effect on TNF-α and IL-1β [6,14,15,16], it is conceivable that this drug may play a beneficial role in the survival and activity of osteoblasts, stimulating ALP and, consequently, favoring the balance in bone turnover disrupted by periodontal disease.

Given the numerous variables that can interfere with the host’s response to bacterial infection during the onset of periodontitis in patients, different animal models have been widely used to investigate this immunoinflammatory disease. Among the animal models, ligature-induced periodontitis in rodents has been considered a useful model for evaluating the complex cascade of cellular and molecular events involved in the progression of the disease [34,35], as well as for evaluating the effectiveness of different therapies in periodontal treatment [6,27,36,37,38,39].

In the present study, we aimed to evaluate whether the inhibition of IL-1β and TNF-α by diacerein could promote bone formation in molars of rats with periodontitis. Therefore, we evaluated whether diacerein treatment interferes with the immunoexpression of OSX and ALP, markers associated with osteoblast differentiation and activity, respectively. Considering that the pro-inflammatory cytokines TNF-α and IL-1β induce osteoblast apoptosis, a possible preventive effect of diacerein on osteoblast death in periodontitis-damaged alveolar bone was also investigated.

## 2. Materials and Methods

### 2.1. Animal Procedures

The study was performed in accordance with Brazilian national care and national laws on animal use. The research protocol was approved by the Ethical Committee for Animal Research of Dental School of Araraquara (CEUA #17/2018 and 12/2020; São Paulo State University—UNESP, São Paulo, SP, Brazil).

In the present study, 117 adult male rats (*Rattus norvegicus albinus*, Holtzman), weighing 220–250 g, were randomly and equally distributed into three groups (*n* = 39 rats per group): control group (CG; healthy periodontium), diacerein-treated periodontitis rats (PDG) and sham-treated periodontitis group (PSG). In each group, 39 rats were euthanized at 7, 15 and 30 days (13 rats per period). The sample size was calculated considering the bone loss as the primary outcome variable, which was estimated in our previous studies [6,27]. This estimation was performed for the detection of a difference of 40% between rats with periodontitis (PDG and PSG) and healthy rats (CG). Considering a 90% test power and an alpha level of 0.05 for recognizing significant differences, at least 6 rats per group in each period were necessary for morphological analysis (paraffin-embedded specimens). In addition, 7 rats were added: 4 rats for obtaining gingiva samples for gene expression analysis (*Nfkb1* and *Bmp2*), and 3 rats for ultrastructural analysis. This study used the same animals from a prior investigation conducted in our laboratory [6]. The rats were maintained in a vivarium under controlled temperature (23 ± 2 °C), humidity (55 ± 10%) and photoperiod (12:12 h light/dark cycle), with water and food provided ad libitum.

The rats of PDG and PSG received an intraperitoneal injection of ketamine hydrochloride (80 mg/kg of body weight—bw) and xylazine hydrochloride (8 mg/kg of bw). Under anaesthesia, a cotton ligature was placed around the cervix of the first upper molars for 7 days to induce periodontitis. After ligature removal, the rats of PDG received 100 mg/kg of diacerein (Artrodar^®^, TRB Pharma, São Paulo—SP, Brazil) daily by gavage, while the rats of PSG received an equivalent volume of sterile saline solution. The rats were treated with diacerein or saline solution for 7, 15 and 30 days. The 100 mg/kg dosage was based on a previous study that comparatively analysed the effects of diacerein doses (10 mg/kg, 100 mg/kg, and 200 mg/kg) on rheumatoid arthritis in rats. The 100 mg/kg diacerein dose inhibited the edema and preserved femoral bone mass, while the 10 mg/kg dose failed to attenuate the deleterious effects caused by arthritis. Furthermore, the highest dose (200 mg/kg) showed similar effects to the 100 mg/kg dose, therefore not justifying its use [40]. In rats with periodontitis, daily administration of diacerein at a dose of 100 mg/kg significantly reduced serum IL-1β levels after 4 weeks of treatment [15] and promoted a significant reduction in the number of osteoclasts in the alveolar bone [6]. Twenty-four hours after the last dose, the rats were euthanized with an overdose of ketamine hydrochloride (240 mg/kg of bw) and xylazine hydrochloride (24 mg/kg of bw).

At 7, 15 and 30 days after the start of treatment, 13 rats per group were allocated to the different analyses: 6 rats were used for light microscopy, while samples of 3 rats were processed for transmission electron microscopy, and 4 rats in each group per period were used for RT-qPCR (Reverse Transcription and Real-Time Polymerase Chain Reaction).

### 2.2. Reverse Transcription and Real-Time Polymerase Chain Reaction (RT-qPCR)

Using a stereoscopic microscope (Wild M7; Wild Heerbrugg, Heerbrugg, Switzerland) at ×12 magnification, the gingiva surrounding the first molars was dissected. Following excision, the gingival samples were immediately immersed in RNA Keeper (Nova Biotecnologia; Cotia, Brazil; code: 14-0002-01) for 24 h at 4 °C. After removing the RNA Keeper, the gingival tissue was stored at −80 °C. RNA extraction was performed using an *ReliaPrep™ RNA Tissue Miniprep System* (Promega Corporation, Madison, WI, USA), and reverse transcription was carried out using a High-Capacity cDNA Reverse Transcription Kit (Applied Biosystems, Foster City, CA, USA; code: 4368814). PowerUp SYBR Green Master Mix (Applied Biosystems, Foster City, CA, USA; A25742) was used as a marker, and the QuantStudio 3 system (Applied Biosystems, ThermoFisher, Waltham, MA, USA; Life Technologies Holdings, Singapore) was used for real-time qPCR analysis. Primer sequences (Table 1) were designed using the Primer3 program based on the University of California Genome Browser (UCSC, Santa Cruz, CA, USA). *β-Actin* was used as a housekeeping control and normalization. Real-time PCR was conducted in duplicate for all samples and genes, and the results were reported as mean ± SD, using the formula DCt = [Ct target gene-Ct housekeeping gene *β-Actin*].

### 2.3. Morphological Analysis Under Stereomicroscope

The specimens used to obtain the gingival mucosa samples for RT-qPCR had their maxillae processed for analysis with a stereomicroscope (Zeiss, Discovery, V8; Oberkochen, Germany). After removal of the gingival mucosa, the maxillae were fixed in 4% formaldehyde for 72 h, washed with tap water for 4 h and placed in 70% ethanol. The soft tissues were removed with the help of a scalpel, the maxillae were dried and, subsequently, were immersed for 5 min in 1% methylene blue. After washing in 70% ethanol, the specimens were dried at room temperature, and the images were captured using a digital camera (AxioCAM, ICc5 Zeiss; Oberkochen, Germany) attached to the Zeiss stereomicroscope.

### 2.4. Histological Procedures

The maxillae for morphological analyses were fixed for 72 h in 4% formaldehyde (prepared from paraformaldehyde) in 0.1 M sodium phosphate (pH 7.2) at room temperature. The fragments of maxilla were decalcified in a 7% ethylenediaminetetraacetic acid (EDTA) containing 0.5% formaldehyde buffered with 0.1 M sodium phosphate at pH 7.2. The decalcification of specimens was performed in a microwave oven (Pelco BioWare^®^ Pro 36500, Ted Pella, Redding, CA, USA) under 300 W irradiation at 26 °C [27]. Decalcified specimens were dehydrated using increasing concentrations of ethanol, cleared with xylene and embedded in paraffin at 60 °C for 18 h. Non-serial sagittal sections (6 µm thick) were stained with haematoxylin and eosin (HE) and Masson’s trichrome, subjected to the TUNEL (Terminal deoxynucleotidyl transferase-mediated dUTP Nick-End Labelling) method and other sections were subjected to immunohistochemical reactions for the detection of TNF-α, IL-1β, IL-10, caspase-3, alkaline phosphatase (ALP) and osterix (OSX).

### 2.5. Histological Analysis of Periodontium and Estimation of the Bone Area in the Furcation Region

From the paraffin-embedded maxillae, five non-serial HE-stained sections of each specimen were used to evaluate the morphological changes promoted by periodontitis, with emphasis on the interdental gingiva located between the first and second molars.

Using sections stained with Masson’s trichrome, the percentage of bone tissue occupied in the interradicular region of the first molar was assessed. To estimate the percentage of bone tissue, two non-serial sections exhibiting the periodontium in the furcation region were captured at x10 objective lens from each maxilla. In this field, using an image analysis software (Image-Pro Express 6.0, Olympus, Tokyo, Japan), the bone area was delimited. From the bone area obtained in the furcation region of each specimen, the percentage occupied in the total area was calculated. Thus, the percentage of bone area in the furcation region was calculated for all specimens (*n* = 6 specimens in each group/period).

### 2.6. TNF-α, IL-1β, Caspase-3 and IL-10 Detection by Immunofluorescence

Sections of maxilla fragments containing the molars were subjected to immunofluorescence for the detection of TNF-α, IL-1β, IL-10 and caspase-3. Dewaxed sections were immersed in 0.001 M sodium citrate buffer (pH 6.0) and heated at 96–98 °C in a microwave oven for 30 min. After a cooling-off period, the slides were washed in 0.05 M Tris-HCl-buffered saline (TBS) at pH 7.2 for 15 min, and the sections were incubated in 2% bovine serum albumin (BSA) in a humid chamber for 20 min at room temperature to block non-specific binding. Afterwards, the sections were incubated in a humid chamber at 4 °C for 16–18 h with the following primary antibodies: mouse anti-TNF-α antibody (Abcam, Cambridge Science, Cambridge, UK; code: ab199013) diluted 1:50, rabbit anti-IL-1β (Abcam, Cambridge Science, UK; code: ab283822) diluted 1:50, mouse anti-IL-10 antibody (Santa Cruz Biotecnology, Dallas, TX, USA; code: sc-365858) diluted 1:100 and rabbit anti-caspase-3 (Abcam, Cambridge Science, Cambridge, UK; code: 4051) diluted 1:100. The sections were washed in TBS and, subsequently, they were incubated for 1 h with Alexa Fluor 488 goat anti-mouse (Abcam, Cambridge, UK; code ab 150113) or goat anti-rabbit antibody (Abcam, Cambridge, UK; code ab 150077) diluted at 1:1000, in a humid chamber protected from light at room temperature. DAPI (NucBlue^TM^ Fixed Cell Ready Probes^TM^, Invitrogen, Carlsbad, CA, USA; code: R37606) was used for nuclear staining. In negative controls, the sections were incubated with non-immune serum in place of the primary antibody.

The immunofluorescence reactions were observed using a fluorescence microscope DM4000 B LED (Leica, Wetzlar, Germany) and the images were captured with a camera DFC-550 attached to the microscope. Immunofluorescence for TNF-α, IL-1β and IL10 was measured in the lamina propria of the gingival mucosa of CG, PDG and PSG specimens in all periods. Photomicrographs of the lamina propria of sections subjected to the immunofluorescence reactions for detection of TNF-α, IL-1β and IL-10 were captured at ×40 objective lens (area of 0.05 mm^2^). Using Leica Application Suite software (LAS 4.3; Leica Microsystems, Germany), the immunofluorescent area for each marker (TNF-α, IL-1β and IL-10) was measured. The immunolabelled areas were measured in a total standardised field of 0.05 mm^2^ per specimen in each period (*n* = 6 per group/period). Thus, the percentage of immunofluorescent area for each marker in the total field (0.05 mm^2^) per specimen was calculated. For each immunofluorescence reaction, the parameters of the software LAS 4.3, including threshold adjustment and color range (hue, saturation, and intensity), were rigorously adjusted in all sections to measure the immunofluorescent areas.

### 2.7. Immunohistochemistry for Detection of ALP and OSX

After deparaffinization and hydration, sections were immersed in 5% hydrogen peroxide to block endogenous peroxidase. After washing with TBS, the sections were immersed in 0.001 M sodium citrate buffer at pH 6.0 and heated at 90–94 °C in a microwave for antigen retrieval for 20 min. After cooling at room temperature, the sections were washed with TBS and incubated for 20 min with 5% BSA to block non-specific binding of antibodies. The sections were incubated overnight in a humidified chamber at 4 °C in the rabbit monoclonal anti-ALP antibody (Santa Cruz Biotechnology, Inc., Dallas, TX, USA; diluted at 1:100; code: sc-30203) or in the rabbit polyclonal anti-OSX antibody (Santa Cruz Biotechnology, Inc., Dallas, TX, USA, diluted at 1:75; code: sc-133871). ALP and OSX antibodies were diluted in a medium prepared with 2% BSA and TBS (1:1). After washing in TBS, the immunoreactions were amplified using a Vectastain^®^ ABC Universal Plus Kit—Peroxidase (Vector Laboratories, Inc., Burlingame, CA, USA; code: PK-8200). The sections were incubated for 40 min at room temperature with a multi-link solution containing biotinylated mouse/rabbit antibodies, washed in TBS and incubated with streptavidin-peroxidase complex for 40 min at room temperature. Peroxidase activity was revealed by 3,3′-diaminobenzidine (DAB, DAKO Corporation, Carpinteria, CA, USA; code: K3468) for 2–3 min. After washing, the sections were counterstained with haematoxylin. In sections used as negative controls, the step of incubation in primary antibodies was replaced by incubation in non-immune serum (Sigma-Aldrich Chemie, Munich, Germany).

The number of osteoblasts exhibiting immunolabelling for ALP and OSX was estimated on the surface of the alveolar process. In each section subjected to the immunohistochemistry reactions for ALP or OSX detection, an image of the first molar showing the roots surrounded by alveolar bone was obtained with a light microscope (BX51, Olympu, Tokyo, Japan) using an objective lens at ×4 magnification. The surface of the alveolar process around the first molar was measured with an image analysis software (Image-Pro Express 6.0, Olympus). Under light microscope (Primo Star; Carl Zeiss AG, Oberkochen, BW, Germany), at ×400, ALP- and OSX-immunolabelled osteoblasts present in the alveolar process surface (previously measured) were computed. Thus, the number of ALP- and OSX-immunolabelled osteoblasts computed in each section was divided by previously measured bone surface length, and, consequently, the number of immunolabelled osteoblasts per millimetre of linear bone surface was obtained. The number of ALP- and OSX-immunolabelled osteoblasts was estimated to be 6 specimens per group in each period.

### 2.8. TUNEL Method

The Apop-Tag Plus Kit (Millipore; Burlington, MA, USA; code: S7100) was used for the detection of DNA breaks following the protocol previously described [7,41]. Sections were deparaffinized, hydrated and washed in 50 mM phosphate-buffered saline at pH 7.2 for 10 min. After treatment with proteinase K solution (20 mg/mL) for 10 min at 37 °C to expose the DNA strands, the sections were immersed in 5% hydrogen peroxide for 20 min to block endogenous peroxidase and then the slides were immersed in an equilibrium buffer (provided for the kit) for 5 min at room temperature. Sections were incubated in terminal deoxynucleotidyl transferase (TdT) at 37 °C in a humid chamber. After 1 h, the slides were immersed in the stop-wash solution at 37 °C for 10 min. Sections were then incubated in the anti-digoxigenin-peroxidase solution in a humid chamber for 30 min. Following the washing, the peroxidase activity was revealed by 3,3′-diaminobenzidine (DAB, DAKO Corporation, Carpinteria, CA, USA; code: K3468) for 2–3 min, and the sections were counterstained with haematoxylin. Negative controls were incubated in a TdT-free-enzyme solution. As a positive control, sections of the involuting mammary gland (provided by the manufacturer of the kit) were subjected to the same protocol.

To estimate the number of TUNEL-positive osteoblasts in the bone surface, 2–3 non-serial sections from each maxilla (*n* = 6 per group/period) were used. The surface of alveolar bone around the first upper molars from the sections subjected to the TUNEL method was measured using an image analysis system (Image Pro-Express 6.0, Olympus, Tokyo, Japan) at ×4 objective lens. On this previously measured surface, the number of TUNEL-positive osteoblasts was computed. The number of TUNEL-positive osteoblasts was divided by bone surface length, and, therefore, the number of TUNEL-positive osteoblasts per millimetre of bone surface was obtained.

### 2.9. Ultrastructural Analysis

Ultrastructural analysis was performed on 3 fragments of maxilla containing the alveolar process around the first molars of each group per period. The maxilla fragments were fixed for 24 h in a mixture of 4% glutaraldehyde and 4% formaldehyde (prepared from paraformaldehyde), buffered with 0.1 M sodium cacodylate at pH 7.2 at room temperature [7,42]. Maxilla fragments were decalcified in 7% EDTA containing 0.5% formaldehyde in 0.1 M sodium cacodylate (pH 7.2). After decalcification, the fragments were sliced, and small samples were obtained. These samples were washed and post-fixed in 1% osmium tetroxide for 1 h, washed in distilled water and immersed in 2% aqueous uranyl acetate for 2 h at room temperature. The samples were washed in distilled water and then dehydrated, treated with propylene oxide and embedded in Araldite. Toluidine blue-stained semithin sections were used to select the regions containing alveolar bone. Ultrathin sections were stained with alcoholic 2% uranyl acetate and lead citrate solution, and examined in a transmission electron microscope (TECNAI G2 Spirit, FEI Company; Hillsboro, OE, USA) of the Biosciences Institute (UNESP, Botucatu, SP, Brazil).

### 2.10. Statistical Analysis

Statistical analysis was performed with GraphPad Prism 9.02 software (GraphPad Software, Boston, MA, USA). Data from immunofluorescence and immunohistochemistry reactions were subjected to the Shapiro-Wilk test to verify the normality and, subsequently, the data were evaluated by two-way analysis of variance (ANOVA) and post hoc Tukey’s test. The accepted significance level was *p* ≤ 0.05. For the mRNA levels of *Nfkb1 and Bmp2*, statistical analysis, the one-way ANOVA and Tukey’s test were used. The accepted significance level was *p* ≤ 0.05.

## 3. Results

### 3.1. Histological Description

**Figure 1:**
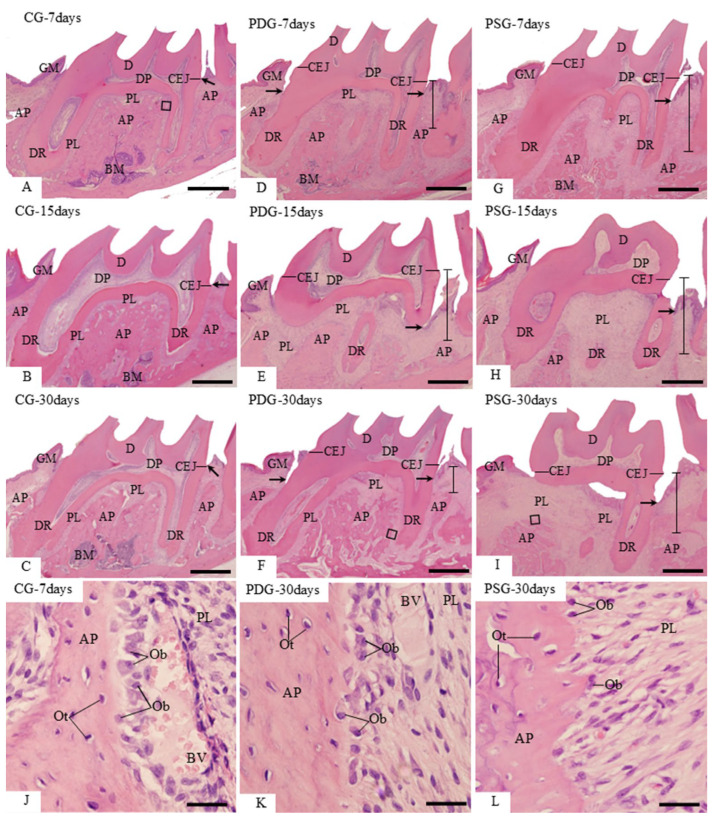
(**A**–**L**): Light micrographs of sagittal sections of first molars stained with HE. (**A**–**I**): general view of first molars surrounded by gingival mucosa (GM), alveolar process (AP) and periodontal ligament (PL). Arrows, junctional epithelium; CEJ, cementum–enamel junction; distance between CEJ and AP is delimited by a line; DR, dental root; BM, bone marrow. (**J**–**L**): high magnification of outlined areas in the (**A**) (CG), (**F**) (PDG) and (**I**) (PSG). (**J**,**K**): show large osteoblasts (Ob) adjacent to the bone surface of the alveolar process (AP). In (**L**), scattered osteoblasts (Ob) are present on the surface of alveolar process (AP). PL, periodontal ligament; Ot, osteocytes; BV, blood vessel. Scale bars: 700 µm (**A**–**I**) and 34 µm (**J**–**L**).

In the CG specimens, the gingiva between the first and second molars showed a pyramidal shape, and the end of the junctional epithelium was situated at the level of the cementum-enamel junction. The periodontal ligament occupied a narrow space between the alveolar process and the outer surface of the dental roots (Figure 1A–C). A continuous layer of large osteoblasts was often adjacent to the bone matrix forming (Figure 1J). In the specimens with induced periodontitis (PDG and PSG), junctional epithelium was situated below the cementum–enamel junction. Specimens of both groups showed an evident bone loss in the furcation region as well as between the first and second molars (Figure 1D–I). Despite the inflammatory cells in the gingival mucosa, in the PDG specimens, particularly at 30 days, some areas of the alveolar process exhibited a continuous layer of large osteoblasts in close contact with the forming bone matrix (Figure 1K). In contrast, in the PSG specimens, osteoblasts were sparsely situated in the irregular bone surface (Figure 1L).

### 3.2. TNF-α and IL-1β Immunofluorescence Findings

**Figure 2:**
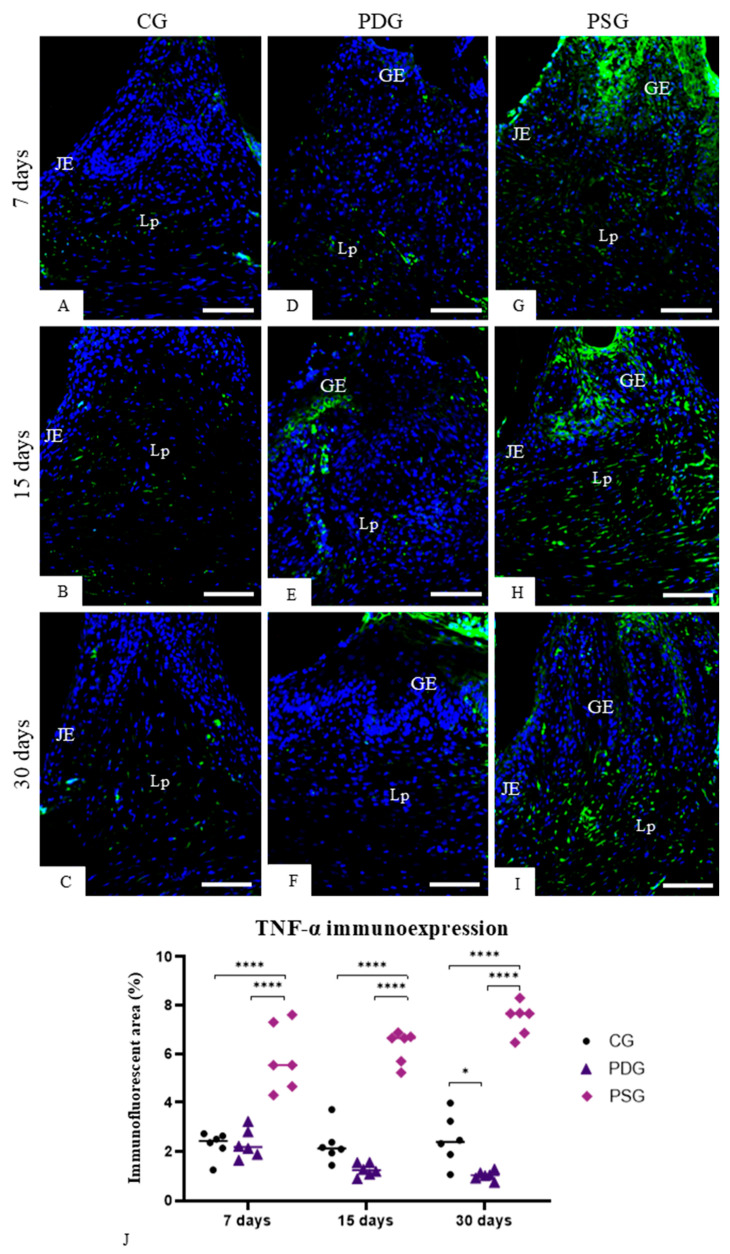
(**A**–**I**): Photomicrographs of sagittal sections of fragments of maxilla showing the gingival mucosa between first and second molars. The sections were subjected to immunofluorescence for detection of TNF-α (green color) and nuclear staining with DAPI (blue color). GE, gingival epithelium; Lp, lamina propria; JE, junctional epithelium. Scale bars: 54 µm. (**J**): TNF-α-immunofluorescent area in the lamina propria of the gingiva. Graphic shows the individual values (dots) and the median of each group (line). Two-way ANOVA followed by Tukey’s test (*p* < 0.05). * *p* < 0.05; **** *p* < 0.0001. *n* = 6 specimens per group in each period.

**Figure 3:**
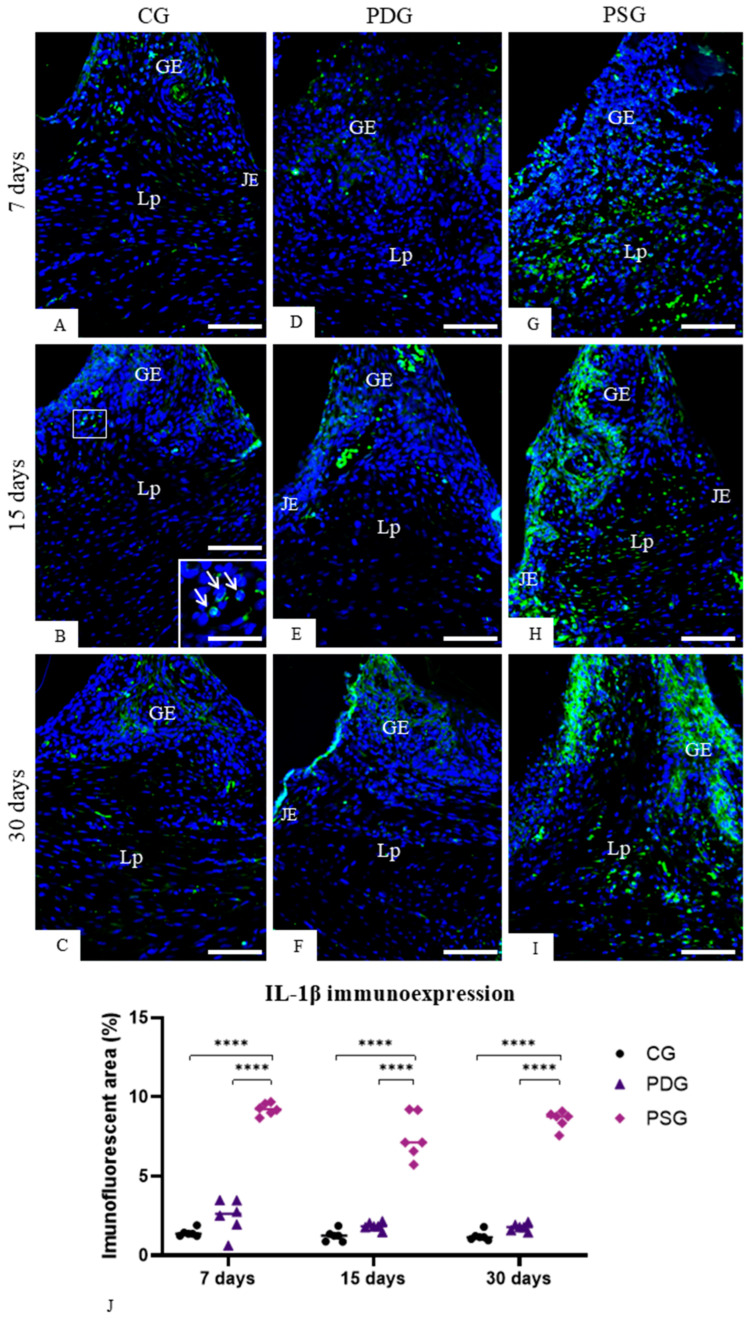
(**A**–**I**): Photomicrographs of sagittal sections of fragments of maxilla showing the gingival mucosa between first and second molars. The sections were subjected to immunofluorescence for detection of IL-1β (green color) and nuclear staining with DAPI (blue color). In (**B**), the outlined area is observed at high magnification in the inset. A strong immunofluorescence is seen in the cytoplasm of some cells (arrows). GE, gingival epithelium; JE, junctional epithelium; Lp, lamina propria. Scale bars: 54 µm and 20 µm (inset of B). (**J**)—IL-1β-immunofluorescent area in the lamina propria of the gingiva. Graphic shows the individual values (dots) and the median of each group (line). Two-way ANOVA followed by Tukey’s test (*p* < 0.05). **** *p* < 0.0001. *n* = 6 specimens per group in each period.

In all periods, few cells in the gingival mucosa exhibited immunolabelling for TNF-α and IL-1β in both epithelium and in the lamina propria of CG (Figure 2A–C and Figure 3A–C) and PDG (Figure 2D–F and Figure 3D–F) specimens. In contrast, a remarkable immunolabelling for TNF-α (Figure 2G–I) and IL-1β (Figure 3G–I) was observed in the gingival epithelium and in the lamina propria of PSG specimens at all time points. The quantitative analysis revealed that PDG specimens contained a lower immunofluorescent area for TNF-α (Figure 2J) and IL-1β (Figure 3J) than PSG specimens (*p* < 0.0001) in all periods. Significant differences in the immunofluorescent area of TNF-α (7 days: *p* = 0.9999; 15 days: *p* = 0.9293 and *p* = 0.6617) and IL-1β (7 days: *p* = 0.6374; 15 days: *p* = 0.9679 and *p* = 0.9894) were not observed between PDG and CG specimens in all periods. In contrast, the immunolabelling for TNF-α and IL-1β was significantly greater in PSG than in CG specimens (*p* < 0.0001) at all periods.

### 3.3. Alveolar Bone Loss in the Furcation Region

**Figure 4:**
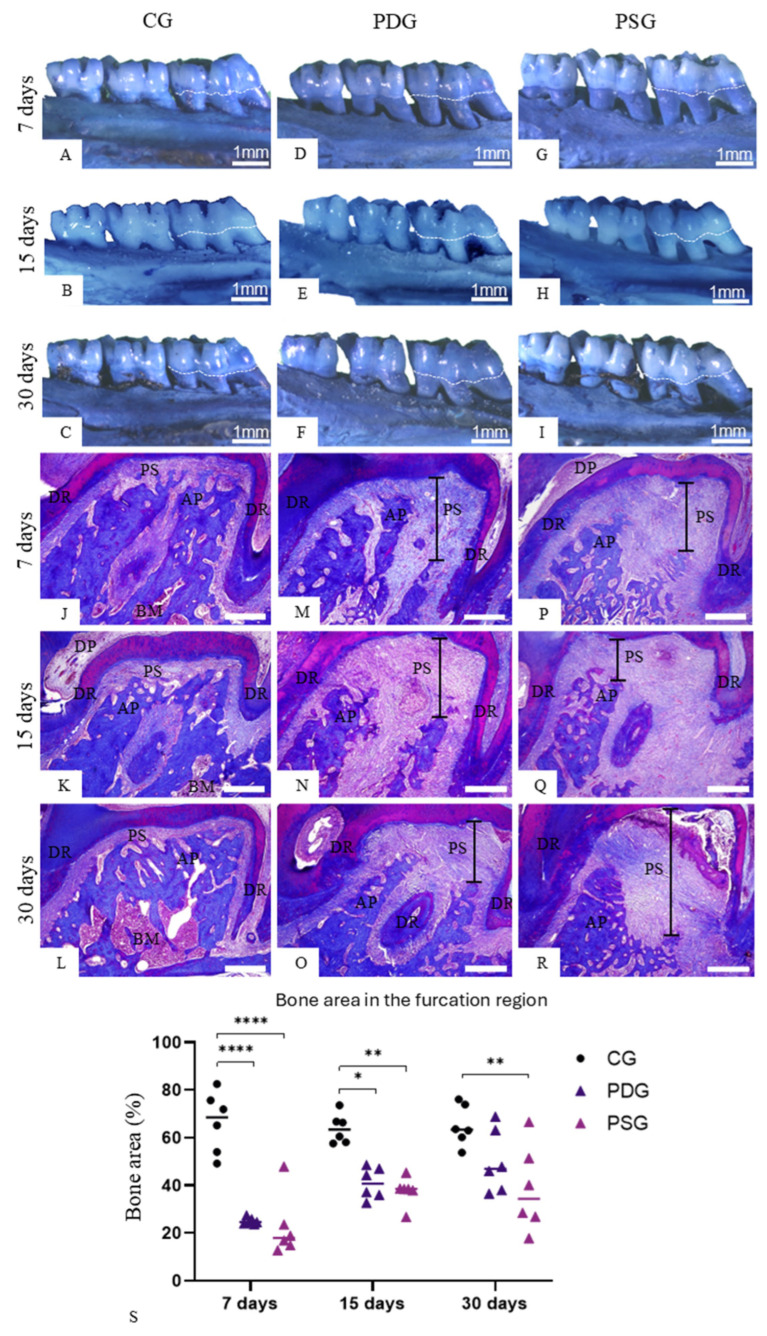
(**A**–**F**): Photomicrographs showing a palatal view of maxillae stained with methylene blue at 7 (**A**,**D**,**G**), 15 (**B**,**E**,**H**) and 30 (**C**,**F**,**I**) days. The hatched line shows the cementum–enamel junction. (**J**–**R**): Light micrographs of portions of first upper molars stained with Masson’s trichrome. General view of furcation region showing the alveolar process (AP) between the roots (DR). (**J**–**L**) (CG): the alveolar process (AP) extends until near the outer root dental (DR) surface; periodontal ligament (PL) fills a narrow space between bone surface and outer root surface. (**M**–**O**) (PDG) and (**P**–**R**) (PSG): alveolar process (AP) exhibiting thin and irregular bone trabecular and thickened periodontal space (PS) is observed. DP, dental pulp; BM, bone marrow. Scale bars: 600 µm. (**S**): Percentage of bone area in the interradicular region. Graphic shows the individual values (dots) and the median of each group (line). Two-way ANOVA followed by Tukey’s test (*p* < 0.05). * *p* < 0.05; ** *p* < 0.01; **** *p* < 0.0001. *n* = 6 specimens per group in each period.

Maxillae stained with methylene blue, analyzed with a stereomicroscope, revealed that PDG (Figure 4D–F) and PSG (Figure 4G–I) specimens had an evident alveolar bone loss in the furcation region in comparison with CG specimens (Figure 4A–C).

Moreover, sections stained with Masson’s trichrome showed that periodontal space was invariably enlarged in the PDG (Figure 4M–O) and PSG (Figure 4P–R) in comparison with CG (Figure 4J–L) at all periods, confirming the bone loss in the interradicular process. Quantitative analysis (Figure 4S) showed that significant differences between PDG and PSG specimens were not observed in the bone area occupied in the interradicular region at all time points (7 days: *p* > 0.9999; 15 days: *p* = 0.9813; 30 days: *p* = 0.7635). However, the analysis over time revealed that there was an increase in the bone area of PDG specimens from 7 to 30 days (*p* = 0.0317). In contrast, no significant difference was detected in PSG specimens over time. Moreover, no significant difference was detected between PDG and CG specimens at 30 days (*p* = 0.4345), while the bone area was significantly lower in PSG than in CG specimens in all time points (7 days: *p* < 0.0001; 15 days: *p* = 0.0026; 30 days: *p* = 0.0108).

### 3.4. Ultrastructural Findings, Caspase-3 Immunofluorescence and TUNEL Method

**Figure 5:**
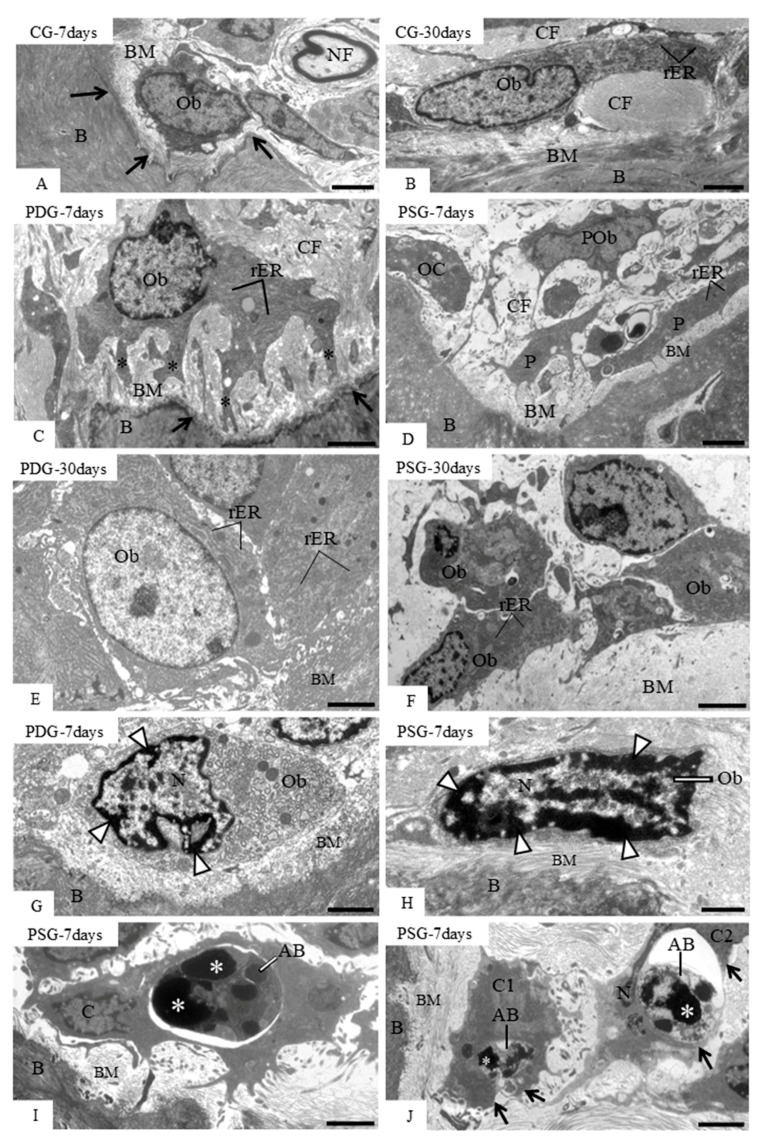
Electron micrographs of portions of alveolar bone (B) of CG (**A**,**B**), PDG (**C**,**E**,**G**) and PSG (**D**,**F**,**H**–**J**). (**A**–**F**): osteoblasts (Ob) and portions of osteoblasts (P) are in close juxtaposition to developing bone matrix (BM). In (**C**), a large osteoblast (Ob) extends cytoplasmic projections (asterisks) towards the excavated bone surface (arrows). In (**D**), an excavated area of alveolar bone (B) exhibits portions of osteoblasts (P) and a portion of an osteoclast (OC). (**E**,**F**): large osteoblasts are seen adjacent to the bone matrix (BM). In (**G**), a large osteoblast (Ob) situated in an excavated bone area (arrows) exhibits an irregular nucleus (N) with peripheral condensed chromatin (arrowheads). In (**H**), an elongated osteoblast (Ob) exhibits a nucleus (N) with irregular masses of condensed chromatin (arrowheads). (**I**): a mononuclear cell (C) near the bone (B) surface contains a round/ovoid body (AB) inside a large vacuole. The round/ovoid body (AB) exhibits electron-opaque structures (asterisks). In (**J**), two round/ovoid bodies (AB) containing strongly electron-opaque material (asterisks) intermingled with granular material are partially surrounded by cytoplasmic processes (arrows) of cells “C1” and “C2”. B, bone; rER, rough endoplasmic reticulum; CF, bundles of collagen fibrils; BM, bone matrix; POb, pre-osteoblast; NF, nerve fiber. Scale bars: 7 µm (**A**,**D**); 5 µm (**B**,**E**,**F**); 4 µm (**C**,**I**); 3.5 µm (**G**,**J**); 2 µm (**H**). *n* = 3 specimens per group in each period.

Analysis of ultrathin sections of CG specimens revealed osteoblasts adjacent to the excavated areas in the bone surface (Figure 5A) or in juxtaposition to the forming bone matrix (Figure 5B). At 7 days, deep excavations in the alveolar bone surface were seen in PDG and PSG specimens (Figure 5C,D). Some of these areas exhibited large osteoblasts with numerous rough endoplasmic reticulum profiles and cytoplasmic projections towards the bone surface; a rich layer of collagen fibrils of the bone matrix was invariably between these osteoblasts and the bone surface (Figure 5C). Sometimes, portions of osteoclasts intermingled with osteoblasts and pre-osteoblasts were seen filling the excavated areas of the bone surface (Figure 5D). At 30 days, the bone surface of PDG (Figure 5E) and PSG (Figure 5F) was mostly covered by large osteoblasts. However, a careful ultrastructural analysis revealed some osteoblasts exhibiting irregular nuclei with condensed peripheral chromatin or with masses of condensed chromatin either in PDG (Figure 5G) or PSG (Figure 5H) specimens. Moreover, round/ovoid bodies exhibiting electron-opaque structures compatible with condensed chromatin were also found on the alveolar bone surface (Figure 5I,J). Some of these round/ovoid structures were apparently inside mononuclear cells (Figure 5I) or partially surrounded by cytoplasmic projections of mononuclear cells juxtaposed to the bone surface (Figure 5J).

**Figure 6:**
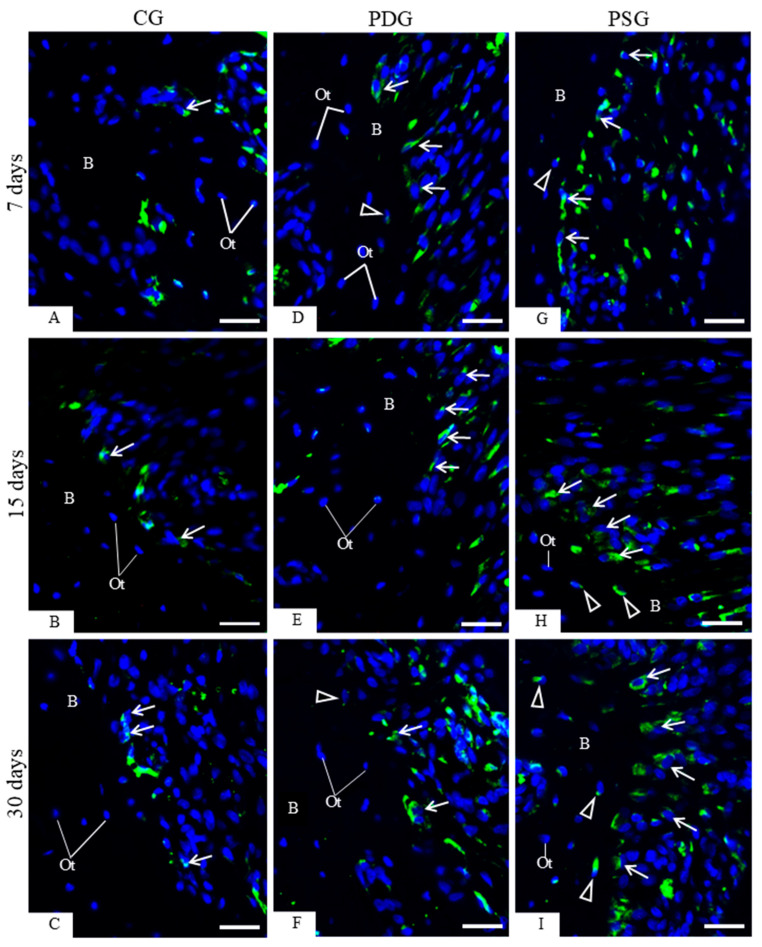
(**A**–**I**): Photomicrographs of sagittal sections of fragments of maxilla showing portions of alveolar bone of the first molar from CG, PDG and PSG specimens. The sections were subjected to immunofluorescence for detection of caspase-3 (green color) and nuclear staining with DAPI (blue color). Osteoblasts (arrows) exhibiting immunolabelled cytoplasm are seen adjacent to the alveolar bone (B) surface. Osteocytes exhibiting immunolabelled cytoplasm for caspase-3 (arrowheads) are observed in PDG and PSG specimens. Ot, osteocytes. Scale bars: 36 µm.

**Figure 7:**
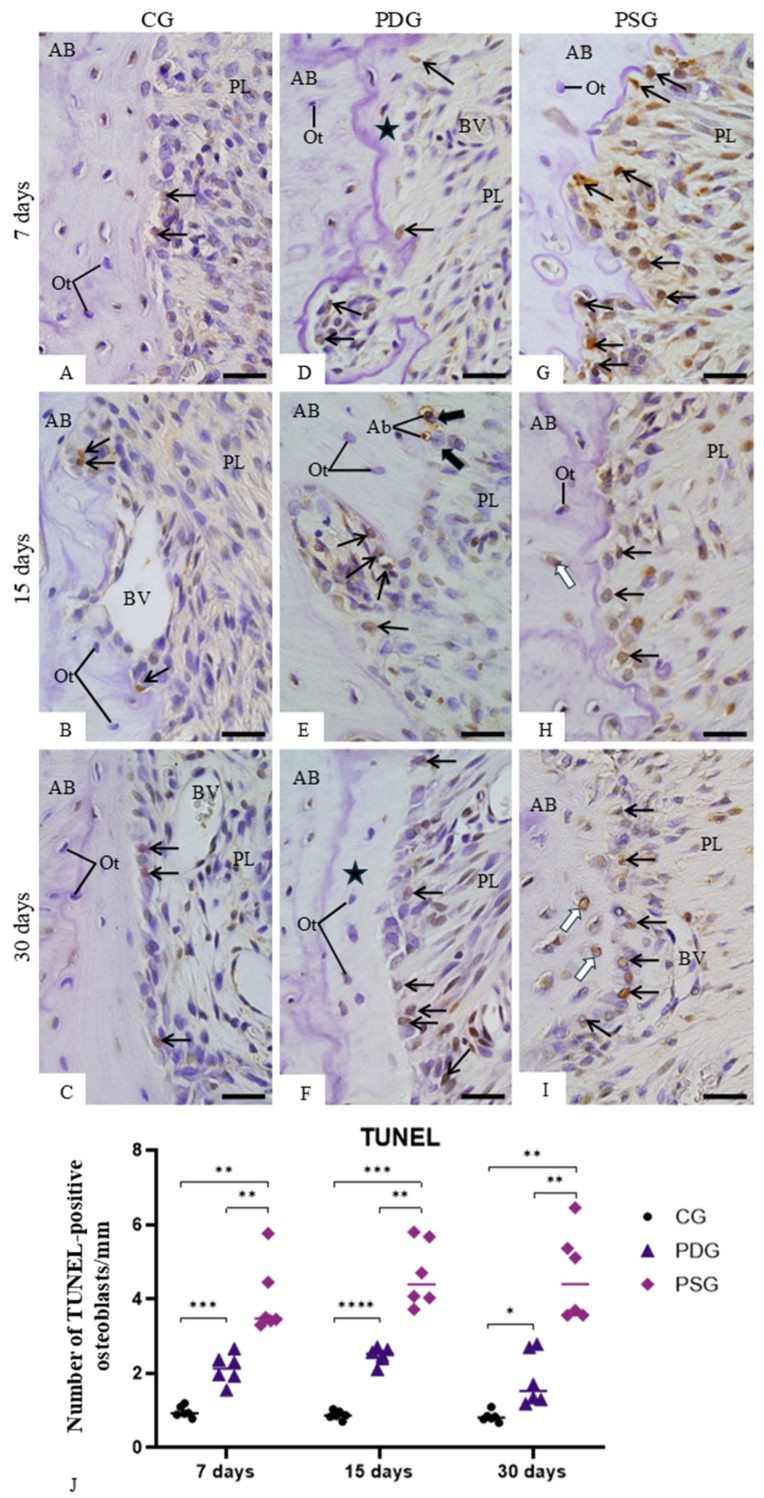
(**A**–**I**)—Light micrographs of sagittal sections of fragments of maxilla showing portions of alveolar bone around the first molar from CG, PDG and PSG specimens. Sections were subjected to the TUNEL method (brown-yellow color) and counterstained with haematoxylin. In CG (**A**–**C**), TUNEL-positive osteoblasts (arrows) are occasionally seen in the alveolar bone (AB) surface. An accentuated number of TUNEL-positive osteoblasts (arrows) is present in the alveolar bone (AB) surface from PSG (**G**–**I**) in comparison with PDG (**D**–**F**) specimens. In (**E**), TUNEL-positive small bodies (AB) appear to be within the vascular structures of mononuclear cells (thick arrows) on the bone (B) surface. Ot, osteocytes; white arrows, TUNEL-positive osteocytes; star, newly formed bone matrix; PL, periodontal ligament; BV, blood vessel. Scale bars: 35 µm. (**J**)—Number of TUNEL-positive osteoblasts in the alveolar bone. Graphic shows the individual values (dots) and the median of each group (line). Two-way ANOVA followed by Tukey’s test (*p* < 0.05). * *p* < 0.05; ** *p* < 0.01; *** *p* < 0.001; **** *p* < 0.0001. *n* = 6 specimens per group in each period.

An accentuated immunoexpression for caspase-3 was observed in the osteoblasts of PSG specimens (Figure 6G–I) when compared with PDG (Figure 6D–F) and CG (Figure 6A–C). Furthermore, osteoblasts exhibiting TUNEL-positive nuclei were occasionally seen among TUNEL-negative osteoblasts in CG specimens (Figure 7A–C). In PDG specimens, few TUNEL-positive nuclei were observed adjacent to the bone surfaces (Figure 7D–F). In contrast, a marked presence of TUNEL-positive osteoblasts was observed in the irregular surface of alveolar bone of PSG specimens (Figure 7G–I), mainly at 7 days (Figure 7G). Apoptotic bodies were also found on the bone surfaces; sometimes, these apoptotic bodies were surrounded by a clear halo, typical of vacuolar structure within the cytoplasm of mononuclear cells (Figure 7E). Quantitative analysis (Figure 7J) revealed that the number of TUNEL-positive cells was significantly reduced in PDG in comparison with PSG specimens at 7 (*p* = 0.0094), 15 (*p* = 0.0033) and 30 (*p* = 0.0030) days. No significant difference was observed in the CG, PDG and PSG specimens over time.

### 3.5. ALP and OSX Immunoexpression

**Figure 8:**
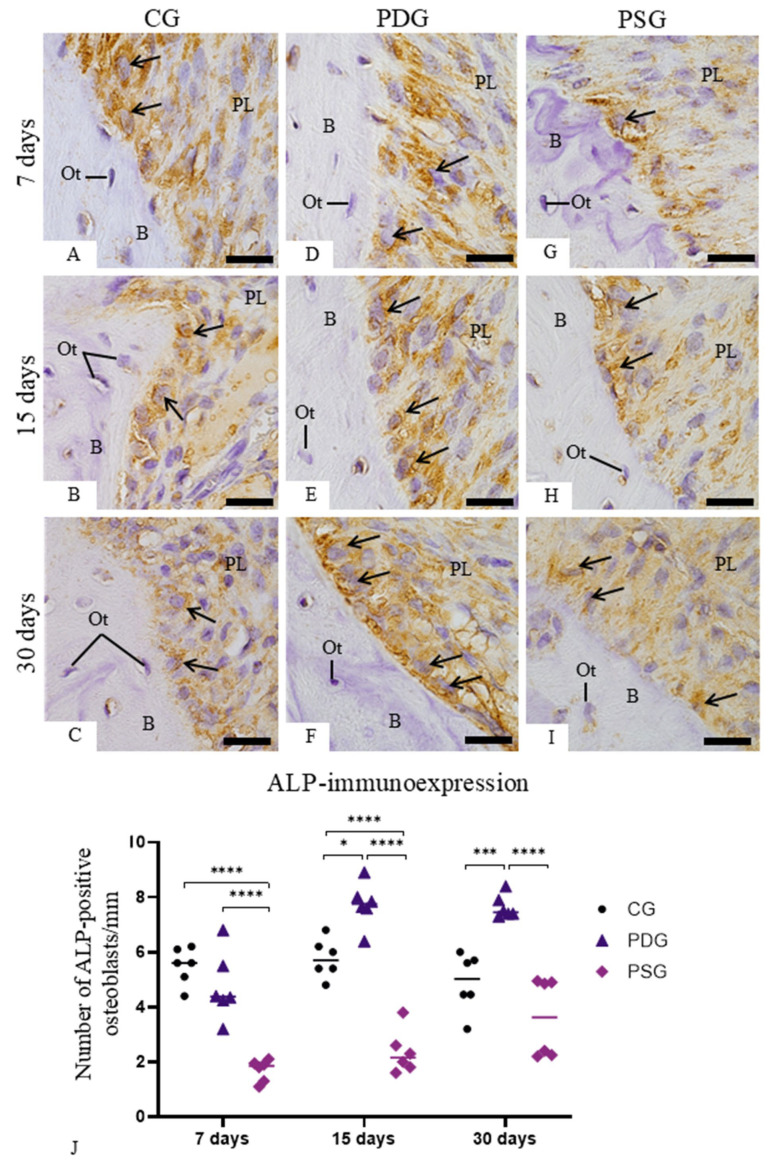
(**A**–**I**): Light micrographs of sagittal sections of portions of maxilla showing the alveolar bone around the first molar from CG, PDG and PSG specimens. The sections were subjected to immunohistochemistry for detection of ALP (brown-yellow color) and counterstained with haematoxylin. ALP-positive immunolabelling is observed in the cytoplasm of osteoblasts (arrows) adjacent to the bone (B) surface. Ot, osteocyte; PL, periodontal ligament. Scale bars: 13 µm. (**J**): Number of ALP-immunolabelled osteoblasts in the bone surface. Graphic shows the individual values (dots) and the median of each group (line). Two-way ANOVA followed by Tukey’s test (*p* < 0.05). * *p* < 0.05; *** *p* < 0.001; **** *p* < 0.0001. *n* = 6 specimens per group in each period.

Osteoblasts juxtaposed to the bone surface of the interradicular alveolar process exhibiting ALP activity were observed in specimens of all groups and time points (Figure 8A–I). However, a different pattern in the immunolabelling was noticed among the groups and time points. A marked cytoplasmic immunolabelling was seen in osteoblasts of PDG mainly at 15 and 30 days (Figure 8E,F) in comparison with CG (Figure 8B,C) and PSG (Figure 8G,I) specimens. As shown in Figure 8J, the number of ALP-immunolabelled osteoblasts was significantly greater in the PDG than in PSG specimens (*p* < 0.0001) at all periods. At 7 days, no significant difference was detected between PDG and CG specimens (*p* = 0.9948), but the number of ALP-immunolabelled osteoblasts was significantly greater in PDG than in CG specimens at 15 (*p* = 0.032) and 30 (*p* < 0.0001) days. In contrast, the PSG had lower values than CG at 7 and 15 days (*p* < 0.0001), although at 30 days, no significant difference was detected between PSG and CG specimens (*p* = 0.830). In CG, no significant difference was found in the ALP immunoexpression over time, while in the PDG, a significant increase was observed in the number of ALP-immunostained osteoblasts from 7 to 15 days (*p* < 0.0001) and from 7 to 30 days (*p* = 0.0008). In PSG, there was a significant increase in the ALP immunoexpression from 7 to 30 days (*p* = 0.0094).

**Figure 9:**
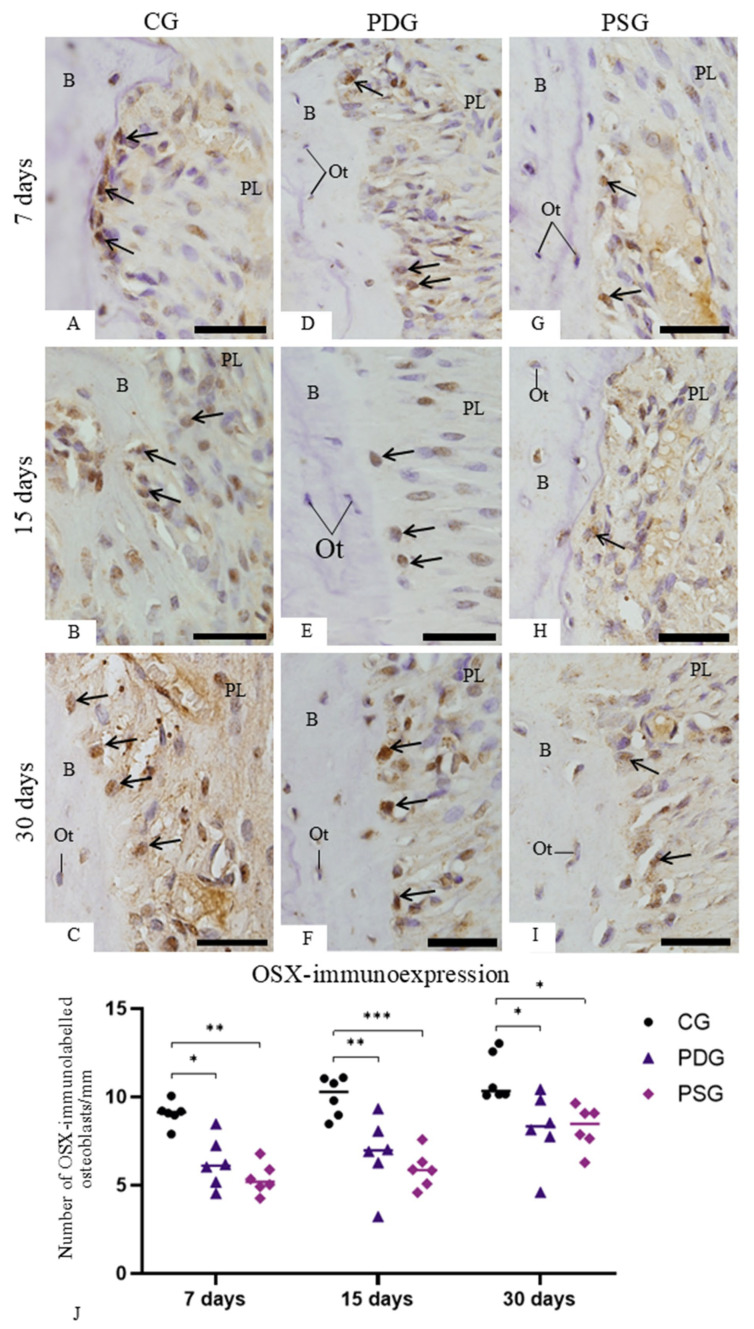
(**A**–**I**): Light micrographs of sagittal sections of fragments of maxilla showing the alveolar bone around the first molar from CG, PDG and PSG specimens. The sections were subjected to immunohistochemistry for detection of OSX (brown-yellow color) and counterstained with haematoxylin. Osteoblasts with OSX-immunolabelled nucleus (arrows) are seen in the alveolar bone (B) surface. Ot, osteocyte; PL, periodontal ligament. Scale bars: 13 µm. (**J**): Number of OSX-immunolabelled osteoblasts in the bone surface. Graphic shows the individual values (dots) and the median of each group (line). Two-way ANOVA followed by Tukey’s test (*p* < 0.05). * *p* < 0.05; ** *p* < 0.01; *** *p* < 0.001. *n* = 6 specimens per group in each period.

Sections of alveolar bone subjected to immunohistochemistry for OSX detection revealed osteoblasts with immunolabelled nucleus (in brown/yellow color) (Figure 9A–I). In the CG (Figure 9A–C) and PDG (Figure 9D–F) specimens, a strong immunoreaction was often found in the osteoblasts located near the excavated bone surfaces and adjacent to the forming bone matrix. In contrast, a weak immunolabelling was present in the osteoblasts in the PSG specimens (Figure 9G–I) in comparison with CG (Figure 9A–C) and PDG (Figure 9D–F). As shown in Figure 9J, no significant difference was detected between PDG and PSG specimens at any time point (7 days: *p* = 0.9664; 15 days: *p* = 0.9620; 30 days: *p* > 0.9999). At all periods, the number of OSX-immunolabelled osteoblasts was significantly lower in PSG and PDG than in CG specimens. Moreover, no significant differences in OSX immunoexpression were observed over time in any group.

### 3.6. IL-10 Immunoexpression in the Lamina Propria of the Gingiva

**Figure 10:**
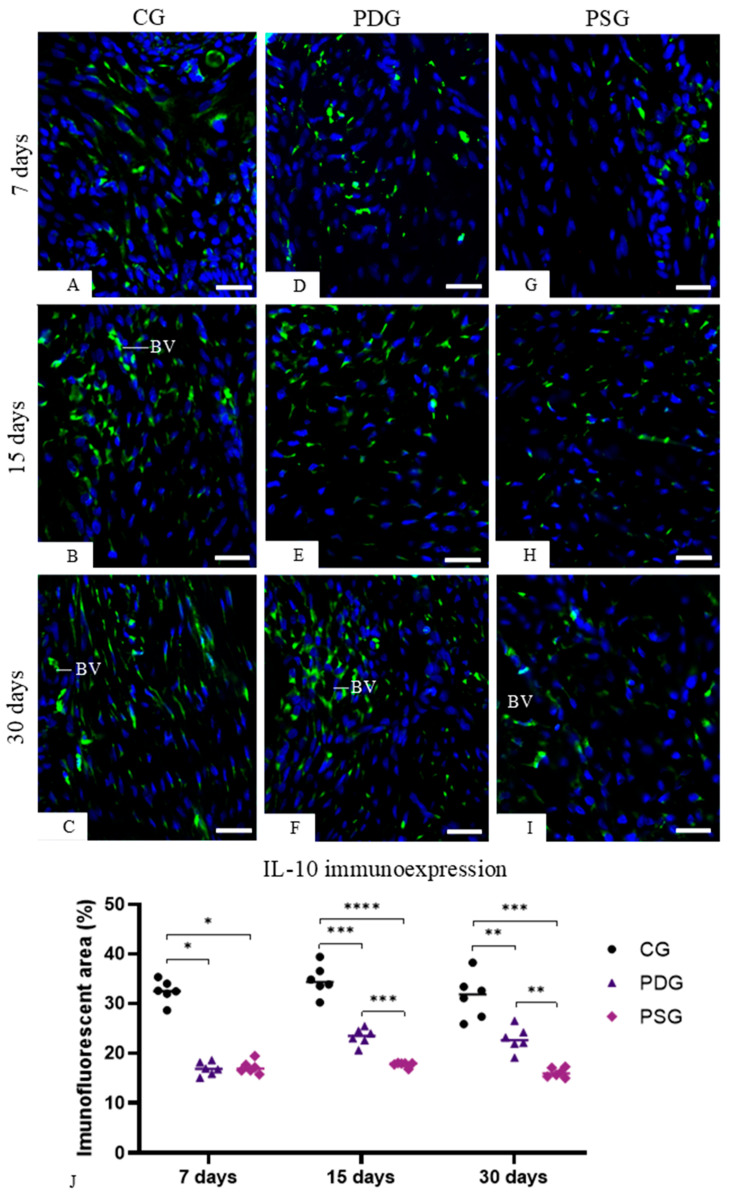
(**A**–**I**): Photomicrographs of sagittal sections of portions of maxilla showing the gingival mucosa between first and second molars. The sections were subjected to immunofluorescence for the detection of IL-10 (green color) and nuclear staining with DAPI (blue color). In all groups, cells with immunopositive cytoplasm are observed in the lamina propria of the gingiva. BV, blood vessel. Scale bars: 36 µm. (**J**): percentage of immunofluorescent area for IL-10 in the lamina propria of the gingival mucosa of CG, PDG and PSG specimens at 7, 15 and 30 days. Graphic shows the individual values (dots) and the median of each group (line). Two-way ANOVA followed by Tukey’s test (*p* < 0.05). * *p* < 0.05; ** *p* < 0.01; *** *p* < 0.001; **** *p* < 0.0001. *n* = 6 specimens per group in each period.

IL-10-immunolabelled cells were observed in the lamina propria of the gingival mucosa of all the groups (Figure 10A–I), although the quantitative analysis showed that the highest values were detected in the CG specimens at all time points (Figure 10J). At 7 days, no significant difference was detected between PDG and PSG (*p* = 0.9296). However, the immunofluorescent area was significantly greater in PDG than in PSG specimens at 15 (*p* = 0.0007) and 30 (*p* = 0.0016) days. The analysis of each group over time revealed that significant differences were not observed in the CG specimens (7 days versus 15 days: *p* = 0.2056; 15 days versus 30 days: *p* = 0.8173; 7 days versus 30 days: *p* = 0.2380). A significant increase in the immunofluorescent areas was observed in the PDG from 7 to 15 days (*p* = 0.0006) and from 7 to 30 days (*p* = 0.0229), but no significant difference was seen from 15 to 30 days (*p* = 0.9237). Significant differences in the IL-10 immunoexpression in the lamina propria of the gingiva were not observed in the PSG over time (7 days versus 15 days: *p* = 0.7157; 7 days versus 30 days: *p* = 0.3558; 15 versus 30 days: *p* = 0.9444).

### 3.7. mRNA Levels of Nfkb1 and Bmp2

**Figure 11:**
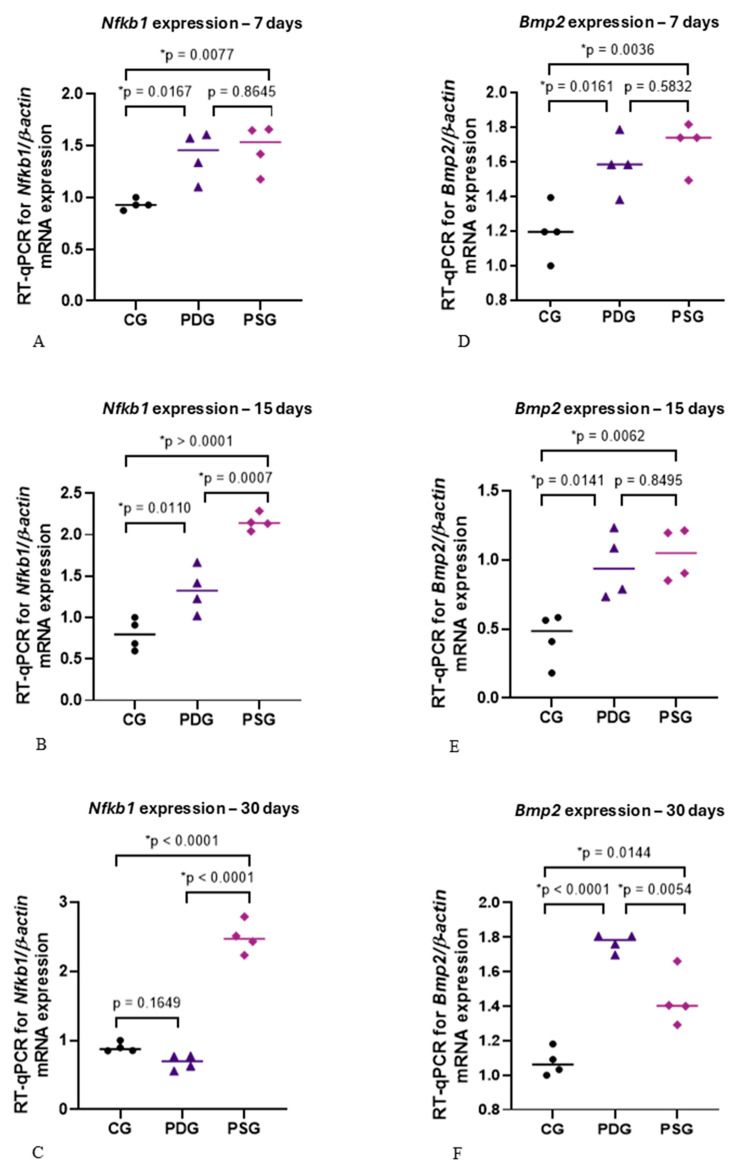
(**A**–**F**): *NfkB* (**A**–**C**) and *Bmp2* (**D**–**F**) mRNA expression levels in CG, PDG and PSG specimens at 7 (**A**,**D**), 15 (**B**,**E**) and 30 (**C**,**F**) days. One-way ANOVA followed by Tukey’s test (*p* < 0.05). *n* = 4 specimens per group in each period. The graphics show the individual values (dots) and the median of each group (line). The following icons represent: • CG; ▲ PDG; ♦ PSG.

Although no significant difference in the mRNA levels of *Nfkb1* was detected between PDG and PSG specimens at 7 days (Figure 11A), *Nfkb1* expression was significantly lower in PDG than in PSG specimens at 15 (*p* = 0.0007) and 30 (*p* < 0.0001) days (Figure 11B,C). At all periods, the *Nfkb1* expression was significantly greater in the PSG specimens than in CG (Figure 11A–C). At 7 and 15 days, the levels of *Nfkb1* expression were significantly higher in the PDG in comparison with CG specimens (Figure 11A,B), but no significant difference was detected at 30 days (Figure 11C).

Regarding *Bmp2* expression (Figure 11D–F), either PDG or PSG exhibited a significant increase in the mRNA levels of *Bmp2* compared with CG specimens at all time points. Significant differences were not observed between PDG and PSG specimens at 7 (*p* = 0.5832) and 15 (*p* = 0.8495) days, but the mRNA levels of *Bmp2* were significantly greater in the PDG than in PSG specimens at 30 days (*p* = 0.0054).

## 4. Discussion

Several in vitro and in vivo studies have demonstrated that TNF-α and IL-1β upregulate RANKL mRNA, promoting osteoclast formation and activity and, consequently, bone resorption [6,43,44]. Diacerein has demonstrated a potent suppressor effect on the mRNA levels of TNF-α and IL-1β in inflammatory diseases such as rheumatoid arthritis [14,16,22] and periodontitis [6]. An evident effect of diacerein on osteoclastogenesis stimulated by periodontitis in rat molars has been observed, promoting a significant reduction in the number of osteoclasts on the alveolar bone surface [6]. Although the effects of TNF-α and IL-1β on osteoclast formation and activity have been widely investigated [6,18,45,46], the effects of these mediators on osteoblast differentiation, activity and survival remain underexplored. Thus, in the present study, we investigated whether diacerein could induce osteoblast differentiation and activity by evaluating the immunoexpression of OSX and ALP. Furthermore, this study assessed whether the inhibitory effect of diacerein on TNF-α and IL-1β could influence osteoblast survival.

Here, diacerein treatment promoted a significant reduction in the immunoexpression of TNF-α and IL-1β in the gingival mucosa of molars with periodontitis, indicating that systemic administration of this drug exerted an effect on the inflamed periodontal tissues. The reduction of these pro-inflammatory mediators in the PDG specimens may be responsible for the increase in the ALP-activity observed in the osteoblasts. Furthermore, diacerein treatment promoted a positive effect on osteoblast survival, as the number of TUNEL-positive osteoblasts was significantly reduced in PDG in comparison with PSG specimens. The significant reduction in the immunoexpression of TNF-α and IL-1β, observed in the gingiva of PDG specimens, reinforces previous findings that demonstrated a reduction in the number of inflammatory cells in diacerein-treated rats with periodontitis [27]. In the present study, our findings revealed that diacerein reduced the marked increase in the levels of mRNA of *Nfkb1* induced by periodontitis. There is evidence that an inflammatory reaction, induced by bacterial infection, leads to an over-activation of NF-κB [18,45]. It has also been suggested that inflammatory cytokines, such as TNF-α and IL-1β, secreted by cells of inflamed tissues promote activation of the transcription factor NF-κB [47,48]. Thus, it is conceivable to suggest that the elevated immunoexpression of TNF-α and IL-β observed in the PSG specimens may be responsible, at least in part, for the increased expression of *Nfkb1* caused by periodontitis at all periods. It is important to emphasize that, in the present study, despite the removal of the ligature, the supragingival and subgingival scaling and root planning were not performed. Therefore, the biofilm adhered to the root surface maintained its potential to induce the host inflammatory response, explaining the elevated immunoexpression of TNF-α and IL-1β found in the PSG specimens. The reduced immunoexpression of these pro-inflammatory cytokines in the gingival mucosa of PDG indicates that diacerein played a modulatory role in the host inflammatory response, mitigating the destruction of periodontal tissues.

After 15 and 30 days of treatment, diacerein was able to promote a significant reduction in *Nfkb1* expression, reinforcing the concept that the increase in TNF-α and IL-1β, as observed in the PSG specimens, may activate the NF-κB factor. High levels of IL-1β activate the NF-κB and MAPK pathways, impairing osteoblast recruitment [49] and differentiation [31]. The significant reduction in the *Nfkb1* expression observed in PDG in comparison with PSG specimens at 15 and 30 days was accompanied by a significant increase in the number of ALP-immunolabelled cells, supporting the concept that NF-κB may modulate the osteoblast activity. Despite the significant increase in *Bmp2* expression at 30 days, combined with increased ALP immunoexpression in osteoblasts in PDG specimens compared to PSG, these results were not sufficient to impact differential bone formation between PDG and PSG specimens. Intriguingly, no significant difference in the bone area of the interradicular process was detected between PDG and PSG specimens at all time points, although a significant increase in the bone area from 7 to 30 days was seen in PDG. Indeed, at 30 days, no significant difference in the bone area was seen between PDG and CG, suggesting that the increase in the ALP immunoexpression in PDG specimens culminated in bone formation. However, it is possible that elevated levels of *Nfkb1* observed at 7 and 15 days in the PDG specimens in comparison with the CG specimens may be responsible for the delay in the bone formation in the diacerein-treated rats with periodontitis. This idea is supported because the *Nfkb1* downregulation induced by diacerein at 30 days was combined with an increase in the bone area in the furcation region of the first molars in PDG specimens.

In the present study, a marked reduction in *Nfkb1* expression was noted in the PDG at 30 days, reaching levels similar to those observed in CG specimens. Therefore, our findings support the concept that high levels of *Nfkb1* inhibit osteoblast activity and bone formation [50]. TNF plays an important role in activating canonical NF-κB signaling, inhibiting the induction of p65 on osteoblast differentiation [32]. Furthermore, the use of S2345, an NF-κB inhibitor, has been shown to promote bone repair and increase bone mineral density in ovariectomized mice [51], reinforcing the premise that canonical NF-κB signaling inhibits bone formation. Here, accentuated immunoexpression of TNF-α was accompanied by marked *Nfkb1* overexpression in the PSG specimens at all time points, indicating that in the inflamed gingiva, the increase in TNF-α may be responsible for *Nfkb1* upregulation, resulting in an inhibitory effect on bone formation. This hypothesis is reinforced by the low ALP immunoexpression observed in PSG compared to PDG at all time points. Additionally, no significant increase in the bone area of PSG specimens was detected over time, indicating that accentuated TNF-α immunoexpression and overexpression of *Nfkb1* inhibit the ALP immunoexpression in osteoblasts, thereby impairing the bone formation. Despite the well-established role of the TNF-induced canonical NF-κB pathway, further studies are needed to corroborate the inhibitory effect of diacerein on *Nfkb1* in periodontitis in rat molars.

It has been demonstrated that BMP-2 induces RUNX2 and OSX overexpression, leading to the differentiation of mesenchymal progenitor cells into osteoblasts [50]. In the present study, periodontitis induced an increase in the *Bmp2* expression in both experimental groups (PDG and PSG) at all periods. However, in the present study, this increase did not stimulate the osteoblast differentiation, as the number of OSX-immunolabelled osteoblasts was significantly lower in the PDG and PSG than in the CG specimens at all time points. Therefore, it is conceivable to suggest that some molecules and/or factors from inflamed periodontal tissues interfered with the complex cascade of events required for protein synthesis following the *Bmp2* gene expression. Further studies are needed to evaluate potential candidates that could block the *Bmp2* pathway, preventing its effectiveness in osteoblast differentiation in the alveolar bone of rats with periodontitis.

It has been demonstrated that elevated concentrations of TNF-α reduce OSX expression and subsequently inhibit ALP activity in mesenchymal stem cells [52]. Therefore, these findings, taken together, indicate that high TNF-α levels may induce *Nfkb1* overexpression, which impairs bone formation as observed in the PSG specimens of the present study. This hypothesis is supported by the fact that a significant reduction in *Nfkb1* expression caused by diacerein observed at 15 and 30 days of treatment was accompanied by a significant increase in ALP immunoexpression in the PDG specimens. Thus, the low expression of *Nfkb1*, concomitantly with high *Bmp2* expression and elevated ALP activity in osteoblasts, may be responsible for the bone formation observed in PDG specimens at 30 days. However, it is important to emphasize that bone remodelling depends on the balance between formation and resorption and, therefore, primarily on the activity of osteoblasts and osteoclasts. Thus, the beneficial effects of diacerein on osteoblast survival and activity observed in the present study may be considered in conjunction with the inhibition of osteoclastogenesis, which resulted in a significant reduction in the number of osteoclasts on the alveolar bone surface of rats with periodontitis, as previously demonstrated [6].

Moreover, the immunoexpression of IL-10 was significantly increased in PDG in comparison to PSG specimens at 15 and 30 days. IL-10 acts as an anti-inflammatory cytokine, which stimulates osteoblast differentiation and suppresses osteoclastogenesis [53]. Recombinant IL-10 proteins added to bone marrow stromal cells (BMSCs) increased the expression of osteogenic-related genes, including *Alp* (alkaline phosphatase), *Col1a* (type I collagen), *Runx2* and *Ocn* (osteocalcin). In addition to the upregulation of these osteogenesis-related genes, after 14 and 21 days of culture, calcium deposits were detected with alizarin red staining, indicating that IL-10 recombinant proteins stimulated the osteogenic differentiation of BMSCs. In contrast, recombinant IL-10 proteins added to bone marrow culture significantly reduced the differentiation of these cells into osteoclasts, supporting the concept that IL-10 suppresses osteoclastogenesis and stimulates osteoblast differentiation [53]. Our results showed that periodontitis significantly reduced the immunoexpression of IL-10 in the gingival mucosa, but diacerein was able to minimize the inhibition caused by pro-inflammatory cytokines, since at 15 and 30 days of treatment, the PDG specimens showed a significant increase in IL-10. A low serum level of IL-10 was also observed in rats up to 21 days after induction of periodontitis with injection of lipopolysaccharide from *Porphyromonas gingivalis* [54], reinforcing the concept that pro-inflammatory cytokines may reduce the production of IL-10. Thus, the reduction in the TNF-α and IL-1β caused by diacerein in the inflamed periodontal tissues may be responsible, at least in part, for the increase in the immunoexpression of IL-10, which in turn stimulated ALP in osteoblasts, as observed in the PDG specimens at 15 and 30 days.

In fact, PDG specimens showed large osteoblasts exhibiting well-developed rough endoplasmic reticulum and Golgi complex, reinforcing the idea that these cells are producing a collagen-rich bone matrix [8]. In contrast, flattened osteoblasts with scarce rough endoplasmic reticulum and Golgi complex were seen in excavated bone surfaces of PSG specimens, particularly at 7 days, indicating that these cells were inactive in the production of bone matrix. Moreover, a marked increase in ALP—an enzyme highly expressed during osteoblast differentiation that plays an important role in bone mineralization [8]—was observed in the PDG specimens. The increased ALP immunoexpression caused by diacerein treatment may be responsible for a subtle but significant increase in the bone area in the furcation region of the first molars found in the PDG specimens over time. There is evidence that increased levels of TNF-α and IL-1β inflammatory mediators inhibit the proliferation of mesenchymal stem cells and their differentiation into osteoblasts [55]. Thus, the marked reduction in the immunoexpression of TNF-α and IL-1β caused by diacerein may have induced ALP activity in osteoblasts, promoting the bone formation in the interradicular alveolar process, as observed at 30 days.

In addition to the inhibitory effect of TNF-α and IL-1β on osteoblast differentiation and activity [56,57], studies have demonstrated that increased levels of these mediators induce apoptosis in osteoblastic cell lines [46,52]. In the present study, the high immunoexpression of these pro-inflammatory cytokines caused by periodontitis culminated in an increase in the number of TUNEL-positive osteoblasts, as observed in PSG specimens. Considering that diacerein promoted a decrease in TNF-α and IL-1β immunoexpression, it is conceivable that the suppression of these pro-inflammatory mediators was responsible for the reduction in osteoblast cell death. This idea is reinforced since an accentuated immunolabelling for caspase-3 was seen in osteoblasts in PSG compared with PDG and CG specimens. Caspase-3, a cysteinyl aspartate-specific proteinase, is an executioner caspase involved in the complex cascade of molecular events that culminate in apoptosis [58]. Activated caspase-3 promotes a series of molecular and morphological changes, such as DNA cleavage, chromatin condensation, cytoskeleton degradation, and the formation of plasma and nuclear membrane blebs [57,59]. Our ultrastructural findings revealed osteoblasts showing nuclei with peripheral condensed chromatin or tortuous masses of condensed chromatin, typical features of cells undergoing apoptosis [7,41,42,60,61,62]. Furthermore, round/ovoid bodies exhibiting electron-opaque structures—with an appearance similar to condensed chromatin and profiles of unaltered organelles—were also seen. This indicates that these bodies may be derived from apoptotic cells, as described in different tissues and organs [7,41,42,60,61]. The presence of TUNEL-positive nuclei and cytoplasmic immunolabelling for caspase-3, associated with ultrastructural features, confirms that the osteoblasts in alveolar bone are undergoing apoptosis. In addition, the cytoplasmic projections of mononuclear cells near the bone surface were seen surrounding these apoptotic bodies, suggesting that they were being engulfed by neighboring cells. In fact, cells undergoing apoptosis express signals which promote macrophage recruitment and/or stimulate the neighboring cells to recognize and internalize apoptotic bodies [7,41]. There is evidence that osteoblasts can internalize and digest apoptotic bodies during the early phase of alveolar bone development [7]. Although apoptotic bodies can be recognized and engulfed by osteoblasts, we cannot exclude the possibility that periodontal ligament macrophages may also take part in this process.

Our findings indicate that the increase in TNF-α and IL-1β caused by periodontitis induces osteoblast apoptosis, reducing bone formation and, consequently, contributing to the imbalance between bone formation and resorption. The inhibitory effect of diacerein on *Nfkb1* expression and TNF-α and IL-1β immunoexpression in inflamed gingiva promoted accentuated ALP immunoexpression by osteoblasts. Furthermore, the significant reduction in the number of TUNEL-positive osteoblasts on the alveolar bone surface found in PDG at all time points indicates that diacerein exerted a beneficial effect on osteoblast survival in periodontitis-induced rat molars. From a translational perspective, our findings suggest that the modulation of the host inflammatory response represents a promising adjunctive strategy for the treatment of periodontitis. By suppressing key pro-inflammatory cytokines such as TNF-α and IL-1β and downregulating NF-κB signaling, diacerein was able not only to attenuate inflammatory burden but also to preserve osteoblast survival and partially restore osteoblastic activity in inflamed periodontal tissues. These effects indicate that targeting inflammatory pathways may contribute to the balance between bone resorption and formation, a critical limitation of conventional periodontal therapy that primarily focuses on bacterial biofilm control. Although mechanical debridement remains the gold standard for periodontitis treatment, host-modulatory agents such as diacerein could potentially enhance clinical outcomes by creating a local microenvironment more permissive to bone formation and periodontal repair.

## 5. Conclusions

Our findings, taken together, indicate that diacerein exerts an inhibitory effect on TNF-α and IL-1β in the inflamed periodontal tissues, reducing *Nfkb1* expression and preventing apoptosis in alveolar bone osteoblasts. Moreover, the diacerein-induced decrease in proinflammatory TNF-α and IL-1β stimulated ALP activity in osteoblasts and enhanced IL-10 production in the inflamed gingival mucosa, culminating in slight bone formation in the alveolar process in the furcation region of the first molars.

## Figures and Tables

**Table 1:** Oligonucleotide primer sequences utilized for RT-qPCR analysis.

Genes	Gene Number NCBI	Number of Bases	Primer Sequence (5′ ⟶ 3′)	MT °C
*β-Actin* (Sigma Aldrich, St. Louis, MO, USA)	NM_031144.3	20 19	(F) ACGGTCAGGTCATCACTATC (R) TGCCACAGGATTCCATACC	59 63.4
*Nfkb1* (Exxtend, Paulínia, Brazil)	NM_001276711.1	20 20	(F) CCTCTCTGTTTCGGTTGCTC (R) TACCCTCAGAGGCCAGAAGA	57 57
*Bmp2* (Exxtend, Paulínia, Brazil)	NM_017178.1	20 20	(F) CCCTCCACAACCATGTCCTG (R) GATGGACAGCACAGGGACAC	60 60

## Data Availability

The original contributions presented in this study are included in the article. Further inquiries can be directed to the corresponding author.
